# Strabismus Surgery in Hemophilia: A Novel Surgical Technique and Guideline Recommendations

**DOI:** 10.7759/cureus.51022

**Published:** 2023-12-24

**Authors:** Mahmoud M Ismail, Ali H Alsaedi, Evelyn A Paysse

**Affiliations:** 1 Ophthalmology, King Abdulaziz Medical City (KAMC), Cairo, EGY; 2 Ophthalmology, King Abdullah Medical City, Mecca, SAU; 3 Ophthalmology, Texas Children's Specialty Care, Texas, USA

**Keywords:** subconjunctival, surgery, strabismus, novel technique, haemophilia

## Abstract

Hemophilia is a serious X-linked inheritance coagulation factor deficiency. Clinically, prolonged bleeding or delayed clotting in any area of vascular disturbance is the main manifestation of all hemophilia. We presented a 23-year-old male with a history of left sensory esotropia since the age of three. The patient had not undergone any previous eye surgery and refused to wear glasses. Hematologic studies confirmed a diagnosis of hemophilia A. Upon ophthalmologic examination, the patient's visual acuity was 20/20 in the right eye and 20/120 in the left eye, with deep amblyopia. The patient exhibited left inferior oblique overaction and a V pattern. The ophthalmologic examination otherwise revealed no abnormalities. Preoperative correction of factor VIII was deemed necessary, and the recommended dose was administered to raise the factor VIII level to 52%. The patient underwent bilateral medial rectus recession, left lateral rectus plication, and left inferior oblique myectomy. A new technique utilizing viscodissection with subconjunctival injection of a viscoelastic solution was employed to minimize intraoperative bleeding, resulting in reduced bleeding compared to standard strabismus surgery. No unusual bleeding occurred during the procedure. No postoperative bleeding was observed. The patient was discharged on the fourth postoperative day, having achieved satisfactory cosmetic alignment in the primary position with no complications related to hemophilia. In conclusion, strabismus surgery can be performed safely in strabismic patients with hemophilia. Viscodissection is a helpful novel surgical technique to decrease the risk of bleeding during surgery, and we recommend using this technique in patients using anticoagulants. A multidisciplinary team approach and strict post-operative monitoring are essential in order to achieve optimal results.

## Introduction

Hemophilia is a serious X-linked inheritance coagulation factor deficiency. It commonly occurs in 1:5-10000 males with no racial predilection [1]. There are multiple subtypes of hemophilia; hemophilia type A (factor VIII deficiency) represents 85% [1]. Clinically, prolonged bleeding or delayed clotting in any area of vascular disturbance is the main manifestation of all hemophilia.

## Case presentation

**Figure 1:**
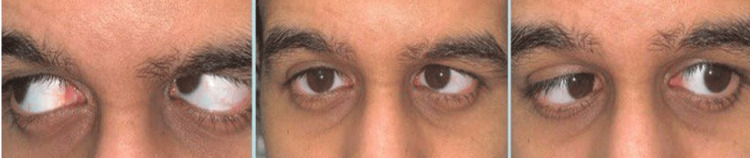
The preoperative presentation of a patient with hemophilia A showed esotropia.

A 23-year-old male presented with left sensory esotropia, which began at age three (Figure 1).

The patient had no history of eye surgery or occlusion and has refused to wear glasses since childhood. The patient has a history of prolonged bruises and ecchymosis after trauma but no active bleeding. Haematologic studies confirmed a diagnosis of hemophilia A with a positive family history (his grandfather on his mother's side). Previous injections of factor VIII were given preoperatively in two procedures before: hand surgery five years ago and dental extraction one year ago. Luckily, there wasn't much bleeding, and the person could lead a regular, active life. He mentioned wanting to correct his eye alignment for work and appearance reasons. The patient entered the hospital a day before the surgery. The examination showed 20/20 vision without glasses in the right eye and (20/120) in the left eye due to a long-standing strabismus. The refraction showed a spherical equivalent of +1.25 in the left eye. The left eye also showed an esotropia of 45 prism diopters for the near primary position, as determined by the Krimsky test. Additionally, there was left inferior oblique overaction and a V pattern. The remainder of the ophthalmologic assessment yielded results within normal limits. Hematologic analysis disclosed a factor VIII level of 8%, indicative of a mild manifestation of hemophilia. No overt joint swelling or other physical manifestations of hemophilia were evident. He was assessed by the hematologist as having moderate-risk bleeding and needed preoperative correction of factor VIII. The recommended dose of factor VIII was calculated as weight x 100 x 0.5 IU and given as a slow intravenous infusion pre-operatively by 1-3 hours, and the factor VIII level reached 52%. The administered dosage was calibrated to elevate the patient's factor VIII concentration from 8% to a deemed safe surgical threshold of 52%. The patient then had a strabismus surgery under general anesthesia, which included a 7 mm bilateral medial rectus recession, an 8 mm left lateral rectus plication, and a 5 mm left inferior oblique myectomy. To decrease the intraoperative bleeding, we created a new technique in the form of viscodissection by subconjunctival injection of viscoelastic solution (sodium hyaluronate) (provisc ͭ ͫ,Alcon) before opening and dissection of the tenon capsule and intermuscular septum. Bleeding was much less than usual during standard strabismus surgery (video 1).

**Video 1:** Viscodissection in strabismus surgery.

**Figure 2:**
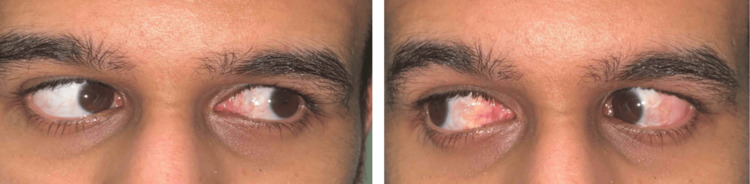
Photos showed the postoperative appearance of the conjunctiva.

No unusual bleeding was encountered during surgery, and no cautery was used during surgery except for the inferior oblique myectomy. After surgery, the patient received three doses of factor VIII, one per 12 hours, guided by factor VIII level. There was no bleeding after the surgery, and the patient was discharged home on the fourth day after the operation. The goal of achieving a pleasing cosmetic alignment in the primary position without hemophilia-related complications was achieved (Figure 2).

## Discussion

There was only one report in the literature about strabismus surgery done on a known hemophiliac. Helveston et al. reported successful strabismus surgery in a 27-year-old known hemophiliac [2]. Strabismus surgery on hemophilia patients is considered a challenge because of the risk of bleeding. In the context of various ophthalmic procedures, the incidence of vision-threatening hemorrhage in strabismus surgery is notably low, albeit recognizing that hemorrhagic complications carry the potential for significant morbidity [3]. Therefore, meticulous surgical planning is imperative to mitigate potential complications. The findings from our study underscore the safe feasibility of strabismus surgery in patients with congenital (factor VIII) deficiency. Guidelines for hemostatic management in hemophilia patients emphasize achieving nearly normal factor levels preoperatively and throughout the perioperative period. On the day of surgery, a bolus of factor VIII concentrate was administered to facilitate safe and bloodless anesthesia procedures, as well as the ensuing strabismus surgery. Careful surgical technique can help minimize intraoperative bleeding. We recommend the injection of viscoelastic solution before conjunctival dissection and at insertion before disinsertion. We noticed that using this method dramatically reduced bleeding and acted as a tamponade to cut vessels. This allowed a good time for the formation of blood clots and decreased the need for cauterization and bleeding in the surgical field. Moreover, this strategy eliminates the requirement for the utilization of agents with alpha-agonist activity, such as topically applied epinephrine and oxymetazoline, traditionally employed to induce vasoconstriction and mitigate intraoperative hemorrhage as tachycardia and hypertension can occur. Viscoelastic material was originally used to maintain a deep anterior chamber during anterior segment surgery. Surgical strategies designed to mitigate intraoperative hemorrhage in hemophilia patients include using viscoelastic injection in all steps of dissection and muscle disinsertion, using blunt dissection to avoid cutting the tenon capsule and intermuscular septum, and using hydrated cotton-tip applicators socketed with balanced salt solution (BSS) for blunt dissection [4]. In addition, employing plication instead of muscle resection and preplacing sutures into the rectus muscle while ligating vessels within the muscle before its disinsertion. Monitoring the patient on the first and second postoperative days to manage any postoperative bleeding [5].

## Conclusions

In conclusion, the performance of strabismus surgery in patients with hemophilia is achievable with a reasonable level of safety. The incorporation of viscodissection, a novel surgical technique, proves beneficial in reducing the risk of bleeding during surgery. We recommend the utilization of this technique, particularly in patients using anticoagulants. To get the best results, it's important to have a multidisciplinary team work together and closely monitor the patient after the surgery.
